# USP43 impairs cisplatin sensitivity in epithelial ovarian cancer through HDAC2-dependent regulation of Wnt/β-catenin signaling pathway

**DOI:** 10.1007/s10495-023-01873-x

**Published:** 2023-12-12

**Authors:** Lipeng Pei, Feng Zhao, Yi Zhang

**Affiliations:** 1 Department of Obstetrics and Gynecology, General Hospital of Northern Theater Command, Shenyang, People’s Republic of China; 2 Department of Stem Cells and Regenerative Medicine, College of Basic Medical Science, China Medical University, Shenyang, People’s Republic of China; 3 Department of Gynecology, The First Hospital of China Medical University, No. 155, Nanjing North Street, Shenyang, People’s Republic of China

**Keywords:** USP43, Epithelial ovarian cancer, HDAC2, Cisplatin sensitivity, Wnt/β-catenin signaling pathway

## Abstract

Epithelial ovarian cancer (EOC) is the leading cause of cancer death all over the world. USP43 functions as a tumor promoter in various malignant cancers. Nevertheless, the biological roles and mechanisms of USP43 in EOC remain unknown. In this study, USP43 was highly expressed in EOC tissues and cells, and high expression of USP43 were associated with a poor prognosis of EOC. USP43 overexpression promoted EOC cell proliferation, enhanced the ability of migration and invasion, decreased cisplatin sensitivity and inhibited apoptosis. Knockdown of USP43 in vitro effectively retarded above malignant progression of EOC. In vivo xenograft tumors, silencing USP43 slowed tumor growth and enhanced cisplatin sensitivity. Mechanistically, USP43 inhibited HDAC2 degradation and enhanced HDAC2 protein stability through its deubiquitylation function. USP43 diminished the sensitivity of EOC cells to cisplatin through activation of the Wnt/β-catenin signaling pathway mediated by HDAC2. Taken together, the data in this study revealed the functions of USP43 in proliferation, migration, invasion, chemoresistance of EOC cells, and the mechanism of HDAC2-mediated Wnt/β-catenin signaling pathway. Thus, USP43 might serve as a potential target for the control of ovarian cancer progression.

**Supplementary Information:**

The online version contains supplementary material available at 10.1007/s10495-023-01873-x.

## Introduction

Ovarian cancer is the second most common cause of gynecological cancer deaths in woman worldwide [1]. Most patients with ovarian cancer are advanced disease at initial diagnosis and relapse within 3 years of diagnosis [2, 3]. Epithelial ovarian cancer (EOC) is the most common pathological type of ovarian malignant tumor accounting for more than 90% [4]. More than 80% of EOC cases have spread to the peritoneal cavity and upper abdominal organs at diagnosis [5, 6]. At present, the main treatment methods include debulking surgery and cisplatin combined with taxane chemotherapy [1]. However, recent studies have shown that patients treated with these compounds develop resistance over time [7, 8]. Therefore, it is of great clinical significance to search for novel therapeutic targets of EOC.

Protein ubiquitination is an important mechanism to regulate protein activity and level under physiological conditions [9]. Loss of ubiquitination regulation of proteins may lead to various diseases, including cancer [10]. Ubiquitin-specific peptidase (USP) is a major member of the ubiquitinase family. Several studies show that USP is essential for cancer progression. USP43 is highly expressed in osteosarcoma and colorectal cancer [11, 12]. USP43 promoted cell proliferation, migration and invasion of colorectal cancer, and reduce the sensitivity of chemotherapy through deubiquitinating ZEB1 [12]. USP43 also exerts a carcinogenic role in breast cancer [13]. However, the role and molecular mechanisms of USP43 during EOC progression are still unknown.

Histone deacetylases control cellular signaling and gene expression. Histone deacetylase 2 (HDAC2) is indispensable for embryonic development and affects cytokine signal transduction related to immune response, and HDAC2 is aberrantly expressed in tumors [14]. HDAC2 plays a carcinogenic role in ovarian cancer, lung cancer, colorectal cancer and other cancers [15–17]. The expression level of HDAC2 increases with the malignant degree of ovarian cancer, and high expression of HDAC2 is related to the poor prognosis of patients [17]. Some studies have shown that USP5 promotes the proliferation of EOC cells by HDAC2 deubiquitylation [18], and silencing HDAC2 increases the sensitivity of ovarian cancer cells to cisplatin [19].

Accumulating evidence has shown that the aberrant activation of the Wnt/β-catenin signaling pathway promotes the development and progression of multiple cancers, including ovarian cancer [20–22]. Inhibition of HDAC2 suppresses the activation of the Wnt/β-catenin signaling pathway [23]. However, the modulation of HDAC2 and Wnt/β-catenin signaling pathways in relation to USP43 remains unclear.

In the current study, we found that USP43 was upregulated in EOC and predicted poor prognosis of patients. USP43 promoted cell proliferation, migration, invasion and chemoresistance of EOC. Further research found that USP43 induced activation of the Wnt/β-catenin signaling pathway by regulating HDAC2. Our study may provide further insight for the EOC at molecular level.

## Methods and materials

### Bioinformatic analyses

Three validated GEO datasets (GSE40595, GSE29450 and GSE38666) were downloaded from GEO database (https://www.ncbi.nlm.nih.gov/geo/). Gene Oncology (GO) functional annotation and Kyoto Encyclopedia of Genes and Genomes (KEGG) pathway analysis of significant differential expression genes were performed using Database for Annotation Visualization and Integrated Discovery bioinformatics resource (DAVID, http://david.abcc.ncifcrf.gov/).

We used GEO2R to analyze the mRNA expression of USP43 in different GEO databases. The overall survival (OS) of patients with EOC was analyzed in the UCSC Xena database (https://xenabrowser.net/).

Protein–protein interaction (PPI) network of USP43 were generated based on the Biogrid database (https://thebiogrid.org/), and the genes in the network were analyzed for GO and KEGG pathway enrichment.

DiseaseMeth web tool (http://bio-bigdata.hrbmu.edu.cn/diseasemeth/) was used to analyze the USP43 methylation level in ovarian serous cystadenocarcinoma (OV) and normal tissues. miRWalk (http://mirwalk.umm.uni-heidelberg.de/) was employed to constructed the miRNA regulatory network with USP43. The protein expression of USP43 in clinical specimens and the survival rate of USP43 in ovarian cancer were analyzed by Human Protein Atlas (https://www.proteinatlas.org/). The correlation between HDAC2 expression and the level of immune cell infiltration was analyzed by TIMER (https://cistrome.shinyapps.io/timer/).

### Immunohistochemistry (IHC)

All tumor samples were formalin-fixed, paraffin-embedded, and sliced into 5 μm sections. The sections were deparaffinized with xylene for 15 min and repaired the antigen with sodium citrate solution. Then the sections were incubated using 3% H_2_O_2_ (Sinopharm) and blocked using 1% Bovine Serum Albumin (BSA, Sangon Biotech). Tumor sections were incubated with the primary antibody diluted in PBS overnight at 4 °C, followed by incubation with the HRP-conjugated goat anti-rabbit secondary antibody (Thermo Fisher Scientific) diluted in PBS for 1 h at 37 °C. The primary antibodies were as follows: USP43 antibody (Affinity Bioscience), HDAC2 antibody (Proteintech Group) and cleaved caspase-3 antibody (Affinity Bioscience). The sections were visualized with DAB solution (MXB Biotech) and counterstained with hematoxylin (Solarbio Science). The stained sections were observed with a microscope (Olympus Corporation).

### Cell culture and infection

HEY and OV-90 cells were purchased from Guangzhou Cellcook Biotech Co., Ltd. HEY cells were cultured in RPMI-1640 medium (Solarbio Science), which included 10% fetal bovine serum (FBS) (Tianhang Biotechnology). OV-90 cells were cultured in DMEM/F12 medium (Biosharp Life Science), which supplemented with 15% FBS. All cells were cultured in in an incubator at 37 °C with 5% CO_2_.

Two USP43 shRNA vectors and USP43 overexpression vector were designed and cloned into transfer plasmids. Lentiviral particles were produced using 293T cells and harvested to infect HEY and OV-90 cells. Puromycin was added into the medium to obtain stably infected cells. For cisplatin sensitivity experiments, cells were treated with the indicated concentrations of cisplatin (meilunbio) for 48 h. To investigate the effect of USP43 on the stability of HDAC2, stable transfected cells were treated with 10 µM cycloheximide (Med Chem Express) for 0, 2, 4 and 6 h, respectively. For the detection of HDAC2 ubiquitination levels, stable transfected cells were pretreated with 20 µM MG132 (Aladdin) for 8 h.

HDAC2 knockdown in HEY cells were carried out using HDAC2 siRNA (si-HDAC2: 5’-GGUCAAUAAGACCAGAUAATT-3’) via Lipofectamine 3000 (Invitrogen), and si-NC as the negative control.

### Quantitative real-time PCR (qPCR)

Total RNA was isolated using a TRIpure commercial kit (BioTeke Corporation). RNA concentrations were determined with the NANO 2000 spectrophotometry (Thermo Fisher Scientific). cDNA was synthesized using reverse transcription with a BeyoRT II M-MLV reverse transcriptase (Beyotime Biotechnology). qPCR was performed with SYBR Green (Solarbio Science) using an ExicyclerTM 96 Real-Time PCR System (Bioneer Corporation). Gene expression was normalized to the expression of GAPDH by the 2^−ΔΔCt^ method. The PCR primer sequences are as follows: USP43 F, 5’-AGGGCTTGAAGAACCACG-3’; USP43 R, 5’-CAGCAACCAGAGCAGGAA-3’.

### Western blot

Total protein was extracted using RIPA buffer (Beyotime Biotechnology) and quantified using a BCA protein assay kit (Beyotime Biotechnology). Equal amounts of protein were separated using SDS-PAGE (Sodium dodecyl sulfate-polyacrylamide gel electrophoresis), then transferred onto PVDF (Polyvinylidene difluoride) membranes (Millipore). After blocking in 5% non-fat milk for 1 h, the membranes were incubated with the primary antibody. The primary antibodies were as follows: USP43 antibody (1: 500 diluted, Santa Cruz Biotechnology), cleaved PARP antibody (1:1000 diluted, Affinity Bioscience), cleaved caspase-3 antibody (1:1000 diluted, Affinity Bioscience), Phospho-Histone H2AX^Ser139^ (γH2AX) antibody (1:1000 diluted, Thermo Fisher Scientific), CDK4 antibody (1:1000 diluted, Affinity Biosciences), CDK6 antibody (1:1000 diluted, Affinity Bioscience), cyclin D1 antibody (1:1000 diluted, Affinity Bioscience), HDAC2 antibody (1:1000 diluted, Proteintech Group), β-catenin antibody (1:1000 diluted, Affinity Bioscience), Ubiquitin antibody (1:1000 diluted, Abclonal Technology), Histone H3 antibody (1:2000 diluted, ABGENT Biotechnology) and GADPH antibody (1:1000 diluted, Santa Cruz Biotechnology). Then the membranes were incubated with the HRP (Horseradish Peroxidase)-conjugated secondary antibody. The secondary antibodies were as follows: goat anti-rabbit antibody (1:5000 diluted, Beyotime Biotechnology) and goat anti-mouse antibody (1:5000 diluted, Beyotime Biotechnology). The protein membranes were visualized by ECL kit (Beyotime Biotechnology).

### CCK-8 and BrdU assays

Cell proliferation was performed with CCK-8 (Beyotime Biotechnology) and BrdU Cell Proliferation ELISA Kit (Abcam) according to the manufacturer’s protocol. Cells were seeded into 96-well plates (4 × 10^3^ cells per well for CCK-8; 2 × 10^4^ cells per well for BrdU), then cultured by CCK-8 or BrdU solution. The absorbance at 450 nm was analyzed with a microplate reader.

For the cell viability assay, cells were plated at a density of 4 × 10^3^ cells per well in 96-well plates and exposed to different concentrations of cisplatin (Aladdin) (0, 5, 10, 50, 100 or 200 µM) for 48 h in 5% CO_2_ at 37 °C. Cell viability was determined by CCK-8 kit (Beyotime Biotechnology) according to the manufacturer’s protocols.

### Colony formation assay

Total of 300 cells were seeded into 60-mm dishes. After 12 days, the culture medium was discarded, and each dish was washed twice with PBS. Then, the cells were stained with Wright-Giemsa stain (Nanjing Jiancheng Bioengineering) according to the attached instructions. Colonies were imaged and counted under the microscope.

### Flow cytometric analysis

Cell cycle analysis was investigated using a Cell Cycle Detection Kit (Keygen Biotechnology) following the manufacturer’s instructions. Briefly, cells were stained with 500 µL PI/RNase A for 30 min at room temperature in the dark. The cells of each period were determined by flow cytometry (Agilent NovoCyte).

For cell apoptosis analysis, cells were treated with cisplatin (HEY: 10 µM, OV-9: 30 µM) for 48 h, cell apoptosis was detected by Annexin V-FITC/PI Apoptosis Detection Kit (Keygen Biotechnology).

### Cell migration and invasion assay

Wound healing assay was conducted to detect the cell migration ability. Cells were cultured in serum-free medium containing 10 µg/ml mitomycin C (Sigma). When the cells reach confluence, gently stroke the center of the culture dish with the tip of a 200-µL pipette. After rinsing with PBS, cells were cultured in serum-free medium for 24 h in 5% CO_2_ at 37 °C. Photographs were taken under the microscope at 0 and 24 h.

Transwell invasion assay was performed in 24-well plates with transwell inserts (Labselect) precoated Matrigel (Corning). Total of 5 × 10^4^ cells (200 µL/well) were placed in top chamber in serum-free medium, and 800 µL culture medium containing 10% FBS was placed in the bottom chamber. After incubating for 24 h at 37 °C in a humidified incubator with 5% CO_2_, the top and bottom chambers were rinsed with PBS, fixed with 4% paraformaldehyde, and stained with 0.4% crystal violet (Amresco) for 5 min. After washing with distilled water, the invasive cells were counted in five randomly microscopic fields.

### Immunofluorescence (IF)

The slides were fixed with 4% paraformaldehyde for 15 min and permeabilized with 0.1% Triton X-100 (Beyotime Biotechnology) for 30 min. After blocking in 1% BSA for 15 min at room temperature, cells were incubated with primary antibody overnight at 4 °C. The primary antibodies were as follows: γH2AX antibody (1:200 diluted, Thermo Fisher Scientific), USP43 antibody (1:50 diluted, Santa Cruz Biotechnology), HDAC2 antibody (1:100 diluted, Proteintech Group) and β-catenin antibody (1:50 diluted, Proteintech Group). Then cells were incubated with Cy3-conjugated goat anti-rabbit antibody (1:200 diluted, Invitrogen), Cy3-conjugated goat anti-mouse antibody (1:200 diluted, Invitrogen) or FITC-conjugated goat anti-mouse antibody (1:200 diluted, Abcam) for 60 min at room temperature in the dark. After washing with PBS, cells were counterstained with DAPI (Aladdin) to mark the nuclei. The slides were sealed with fluorescent mounting medium (Solarbio Science), and observed under the microscope.

### Co-immunoprecipitation (Co-IP)

Co-IP was performed using a Pierce Co-Immunoprecipitation kit (Thermo Scientific Pierce) according to the manufacturer’s instructions. In short, whole proteins were extracted in IP lysis buffer (Beyotime Biotechnology). HDAC2, USP43, ubiquitin and IgG antibodies were pre-incubated with AminoLink Plus coupling resin for 2 h. The resin was washed six times with washing buffer, and incubated with the whole protein lysates overnight. After incubation, the resin was again washed and protein was eluted using elution buffer. Subsequently, the immunoprecipitated protein complexes were collected for Western blot.

### Xenograft tumor models

To assess the effect of USP43 on tumor progression in vivo, we established a xenograft tumor model. The cells (5 × 10^6^) were subcutaneously injected into BALB/c nude mice (17–22 g). After 7 days inoculation, we randomly assigned the mice into four groups which were intraperitoneal injected with saline (Minkang Pharmaceutical) or cisplatin (2 mg/kg) twice a week. Tumor volume was measured every three days starting from day 7. After 4-weeks cisplatin-treatment, tumors were removed, imaged, weighed and pathologically examined.

### Statistical analysis

All data were shown as mean ± SD. GraphPad Prism 8 was used for statistical data in this study. Statistical significance between two sample groups was calculated using t-test. While for comparisons between three or more groups, one-way ANOVA were used, followed by the Tukey post hoc test for multiple comparisons. Two-way ANOVA was performed for group comparisons influenced by two independent factors. *P* < 0.05 was considered significantly different.

## Results

### USP43 is highly expressed in EOC tissues and predicts poor prognosis

**Fig. 1:**
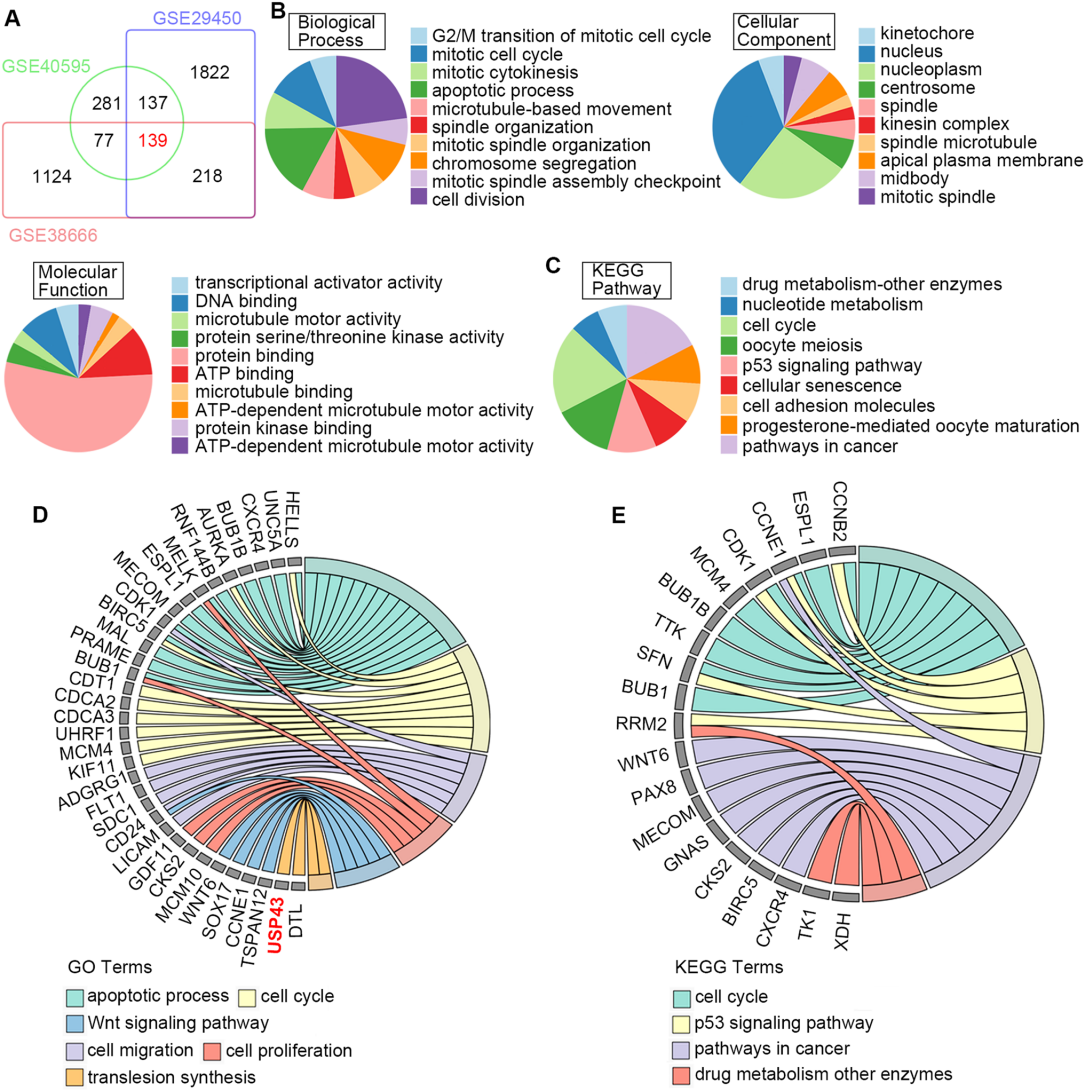
The identification of differentially expressed genes in ovarian cancer and enrichment analysis. **A** Total of 139 common upregulated genes in ovarian cancer tissues were identified in three GEO datasets (GSE40595, GSE29450 and GSE38666). **B, C** GO and KEGG enrichment analysis were conducted for the 139 upregulated genes. **D**, **E** Genes were enriched in GO terms and KEGG pathways related to biological process of ovarian cancer. The right side of the circle shows the enriched genes and the color on the left side of the circle represents the different GO terms or KEGG pathways

Bioinformatics analysis based on the three datasets from GEO database showed that 139 overlapping genes were upregulated in ovarian tissue (Fig. 1A). To understand the potential functions and associated pathways of these overlapping genes, GO and KEGG analyses were conducted. As shown in Fig. 1B, the top enriched GO terms included cell division, mitotic cell cycle, apoptotic process (biological process), nucleus (cell component) and protein binding (molecular function). Pathways in cancer, cell cycle and p53 signaling pathway were the most commonly identified underlying pathways by KEGG (Fig. 1C). Further, the enrichment biological process or pathways that associated with tumor progression was shown in Fig. 1D and E.

**Fig. 2:**
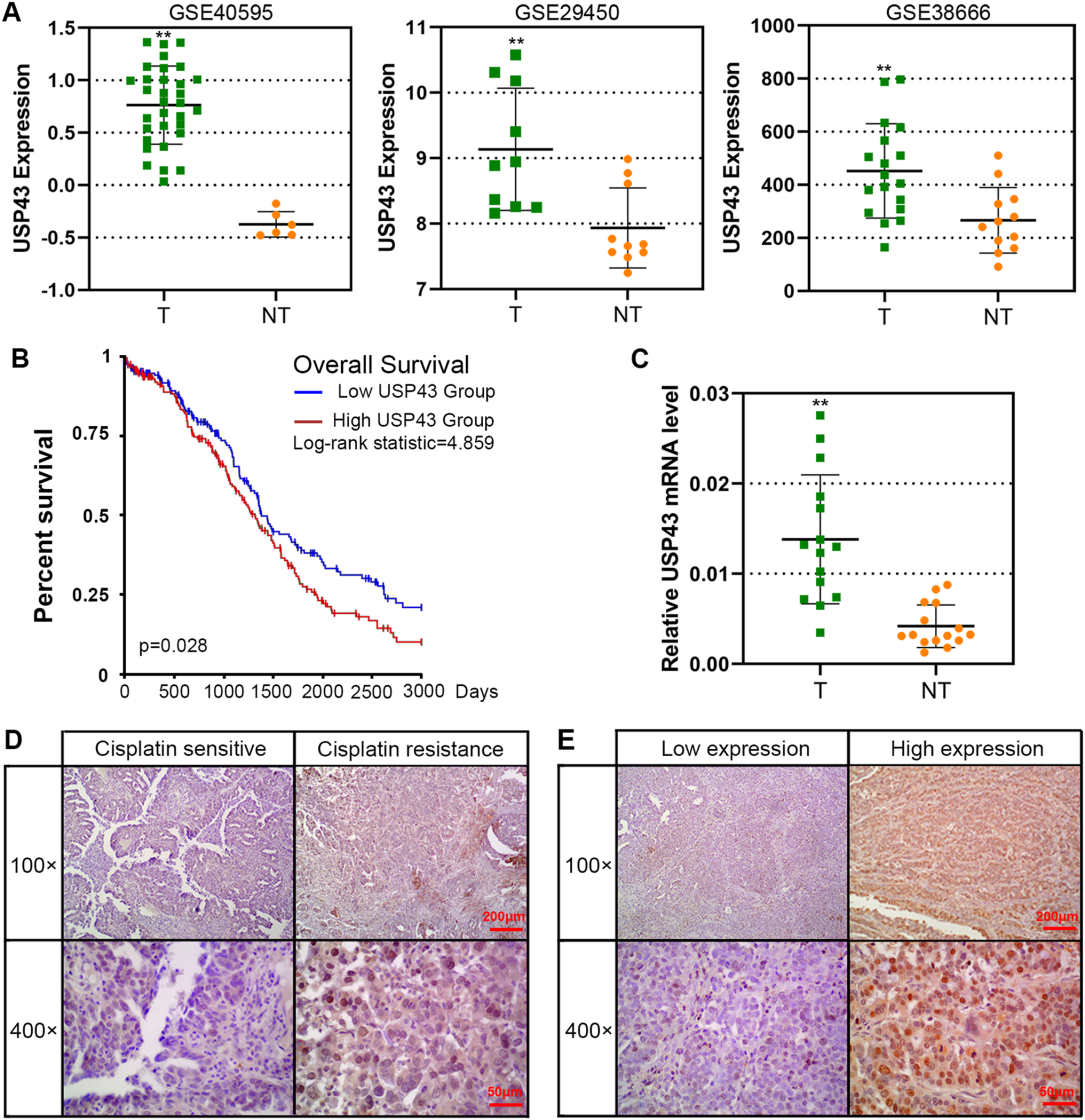
USP43 was upregulated in ovarian cancer tissues. **A** USP43 expression in tumor tissues (T) and normal tissues (NT) were analyzed in three GEO databases (GSE40595, GSE29450 and GSE38666). **B** Kaplan-Meier survival curve evaluating the overall survival of ovarian cancer, based on the expression of USP43. **C** The mRNA levels of USP43 were determined by qPCR in tumor tissues (T) and matched non-cancerous tissues (NT) of 15 EOC patients. **D** Representative images of IHC staining with USP43 in cisplatin-sensitive and cisplatin-resistant EOC tissues. Scale bar, 200 μm or 50 μm. **E** Representative images of IHC staining for high and low expression of USP43 in EOC tissues. Scale bar, 200 μm or 50 μm. ^**^P < 0.01 versus NT

Based on the above analysis and recent studies, we found that USP43 was upregulated and associated with cancer process, but its function in EOC is not understood. Therefore, further experimental studies on USP43 are needed. As shown in Fig. 2A, the upregulated mRNA expression level of USP43 in EOC was confirmed in the three GEO databases. Furthermore, the high expression of USP43 predicted a poor prognostic of ovarian cancer patients (Fig. 2B). qPCR analysis revealed that the mRNA expression level of USP43 in tumor tissues (T) was significantly higher than in adjacent normal tissues (NT) of EOC patients (Fig. 2C). USP43 expression was higher in tumor tissues of cisplatin-resistant patients than in those sensitive to cisplatin (Fig. 2D). The representative images for low or high expression of USP43 were presented in Fig. 2E. These data demonstrated that USP43 was highly expressed in the EOC, and indicated a poor prognosis of EOC.

### USP43 exerts a carcinogenic effect in EOC cells

**Fig. 3:**
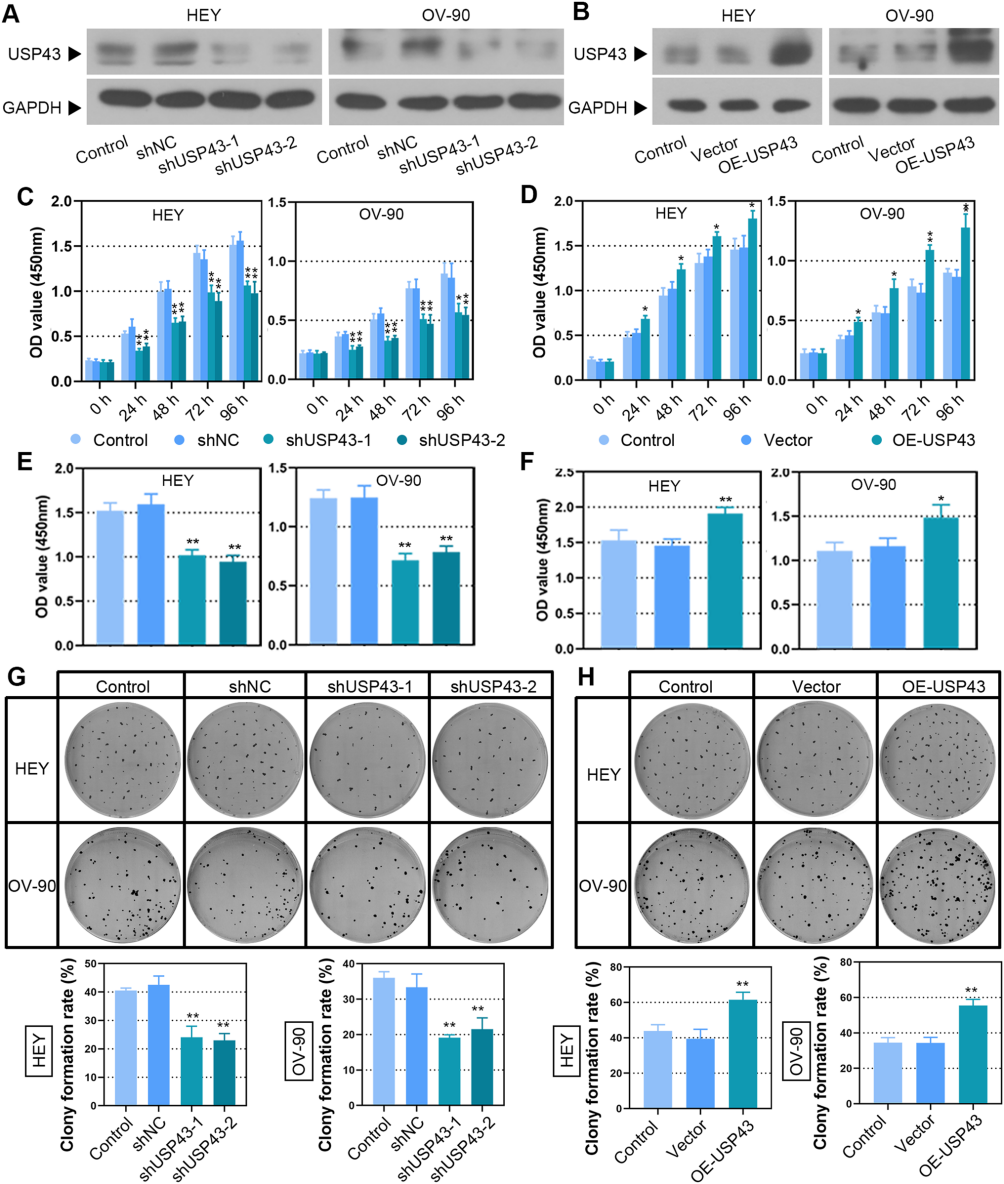
USP43 promoted EOC cell proliferation and colony formation. **A, B** USP43 expression was detected by Western blot in HEY and OV-90 cells with or without USP43 knockdown or overexpression. **C, F** Cell proliferation was detected by CCK-8 assay (**C**, **D**) and BrdU incorporation **E, F**. **G, H** Representative colony formation assays for HEY and OV-90 cells (upper panel); quantitative analysis of colony formation assays (lower panel). ^*^P < 0.05, ^**^P < 0.01 versus shNC or vector

**Fig. 4:**
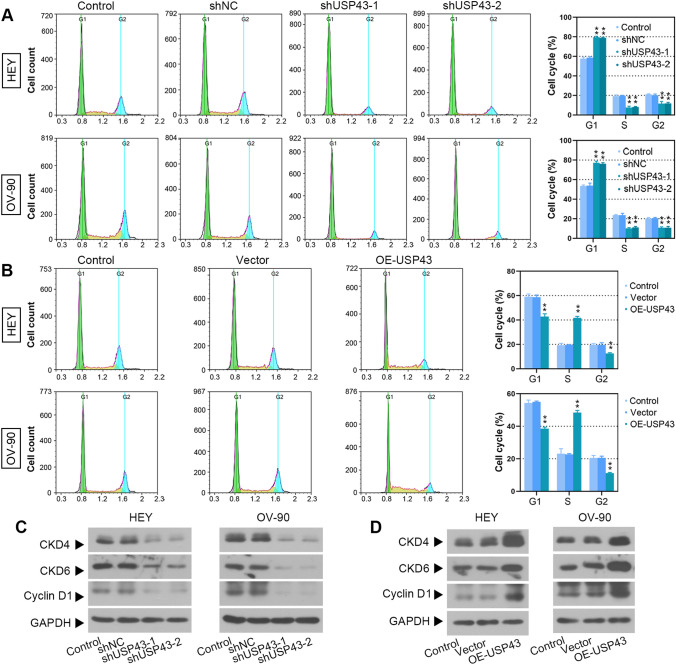
USP43 accelerated cell cycle progression of EOC. **A, B** Cell cycle distribution was detected in HEY and OV-90 cells by flow cytometry (left panel); cells were counted for each phase (right panel). **C, D** CDK4, CDK6 and Cyclin D1 protein expression levels were measured by Western blot. ^**^P < 0.01 versus shNC or vector

**Fig. 5:**
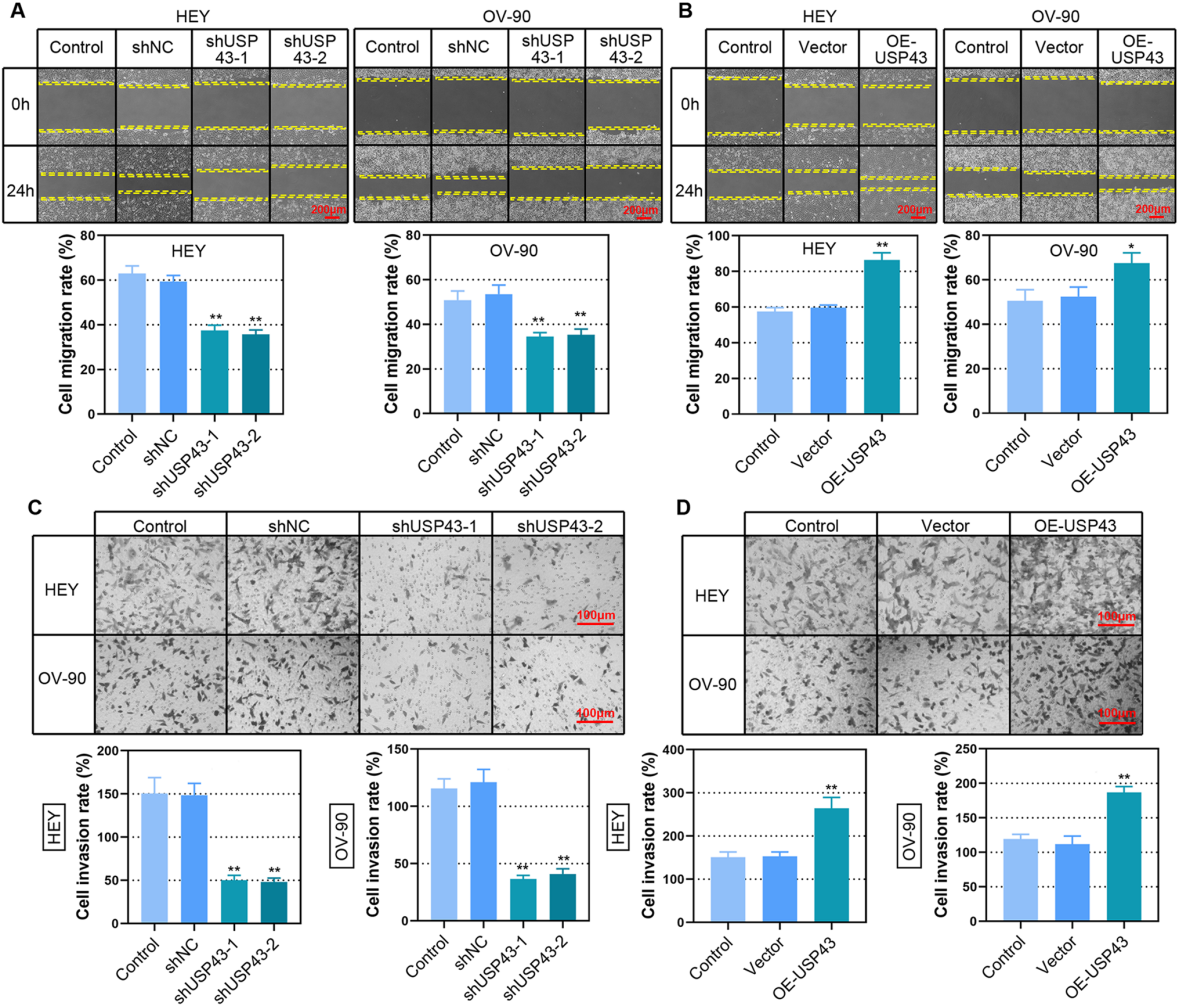
USP43 enhanced the ability of migration and invasion of EOC cells. **A, B** Cell migration ability was assessed with the wound healing assay. Representative photographs of cell migration (upper panel); cell migration rate was analyzed (lower panel). Scale bar, 200 μm. **C, D** Transwell assay was used to assess cell invasion ability. Representative photographs of cell invasion (upper panel); analysis of cell invasion rate (lower panel). Scale bar, 100 μm. *P < 0.05, **P < 0.01 versus shNC or vector

To investigate the biological effects of USP43 on EOC, we evaluated the function of USP43 in EOC cell proliferation, clonogenicity, cell cycle, migration and invasion. First, USP43 was effectively overexpressed or knocked down in EOC cells as confirmed by Western blot (Fig. 3A and B). Decreased proliferation of USP43 knockdown EOC cells was observed by CCK-8 and BrdU assays (Fig. 3C and E). Overexpression of USP43 significantly increased cell proliferation (Fig. 3D and F). Moreover, USP43 silencing repressed the growth of EOC cells (Fig. 3G), while USP43 overexpression exerted the opposite effects (Fig. 3H). The percentage of EOC cells in G1-phase was significantly increased after USP43 knockdown, with a concomitant decrease in the percentage of EOC cells in S-phase and G2-phase (Fig. 4A). USP43 overexpression was observed to induced a decrease in G1-phase and G2-phase cells and an increase in S-phase cells (Fig. 4B). In addition, the expression of CDK4, CDK6 and cyclin D1 was downregulated upon USP43 silencing (Fig. 4C) and upregulated by USP43 overexpression (Fig. 4D), which contributes to cell entry into the proliferation cycle. Furthermore, wound healing and transwell assays demonstrated that the ability of migration and invasion was decreased in USP43-silenced EOC cells (Fig. 5A and C). Whereas USP43-overexpressed EOC cells showed an enhanced migration and invasion ability (Fig. 5B and D). In summary, these findings suggested that USP43 may exerts a carcinogenic effect in EOC cells.

### USP43 impaired cisplatin sensitivity of EOC cells in *vitro*

**Fig. 6:**
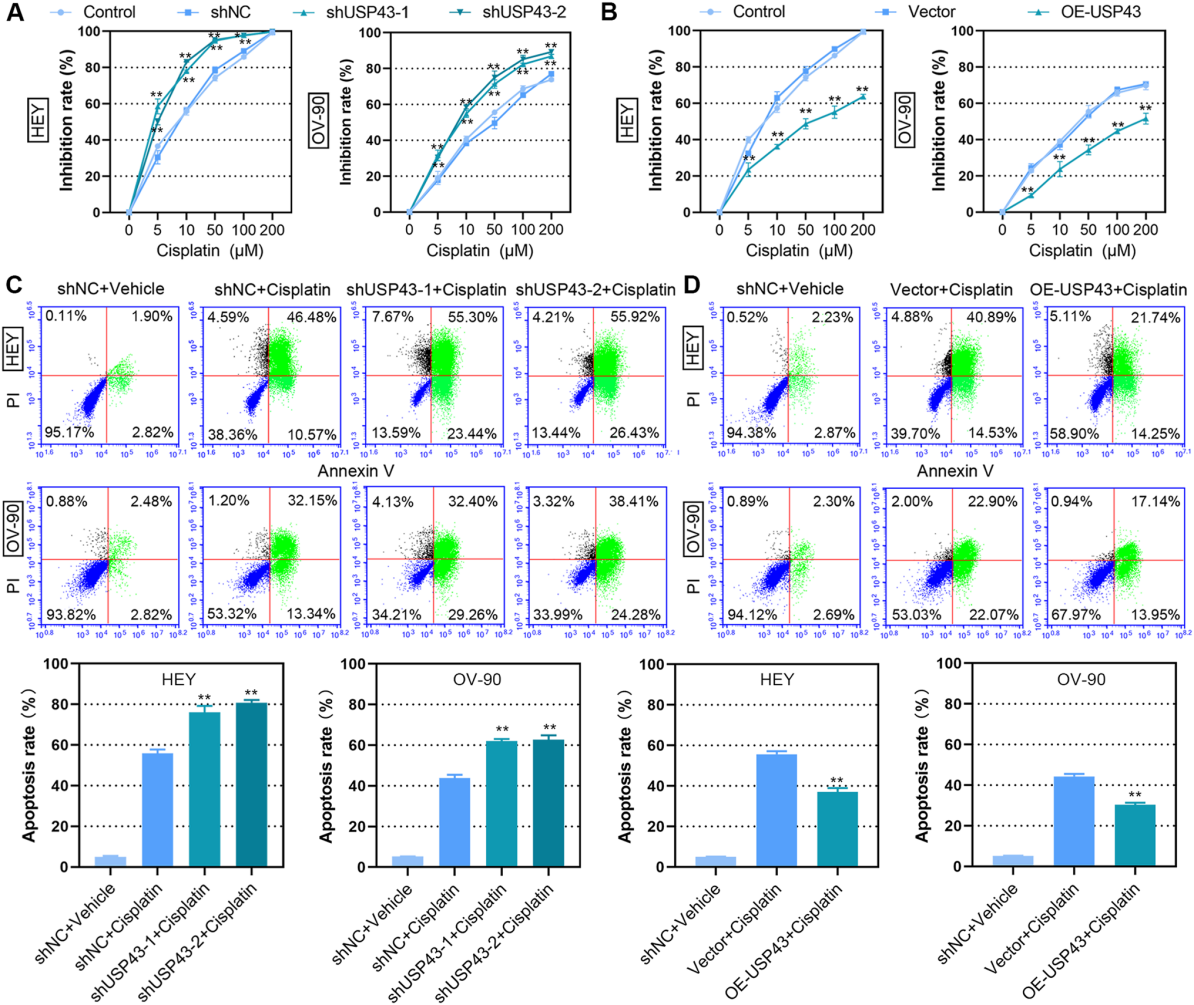
USP43 suppressed cisplatin sensitivity of EOC cells. **A, B** CCK-8 was used to detect cell viability after treatment with different concentrations of cisplatin. **C**, **D** Apoptotic cells were measured by flow cytometry and the apoptosis rate was calculated. **P < 0.01 versus shNC, vector or shNC + cisplatin

To determine whether USP43 was involved in drug sensitivity for EOC treatment, we first measured the inhibition effect of cisplatin on EOC cells with USP43 overexpression or knockdown. The inhibition rate in USP43-silenced cells was increased dose-dependently (Fig. 6A). Cells overexpressing USP43 showed decreased sensitivity to cisplatin (Fig. 6B). Notably, knockdown of USP43 enhanced cisplatin-induced apoptosis (Fig. 6C), while USP43 overexpression diminished the effect of cisplatin (Fig. 6D).

**Fig. 7:**
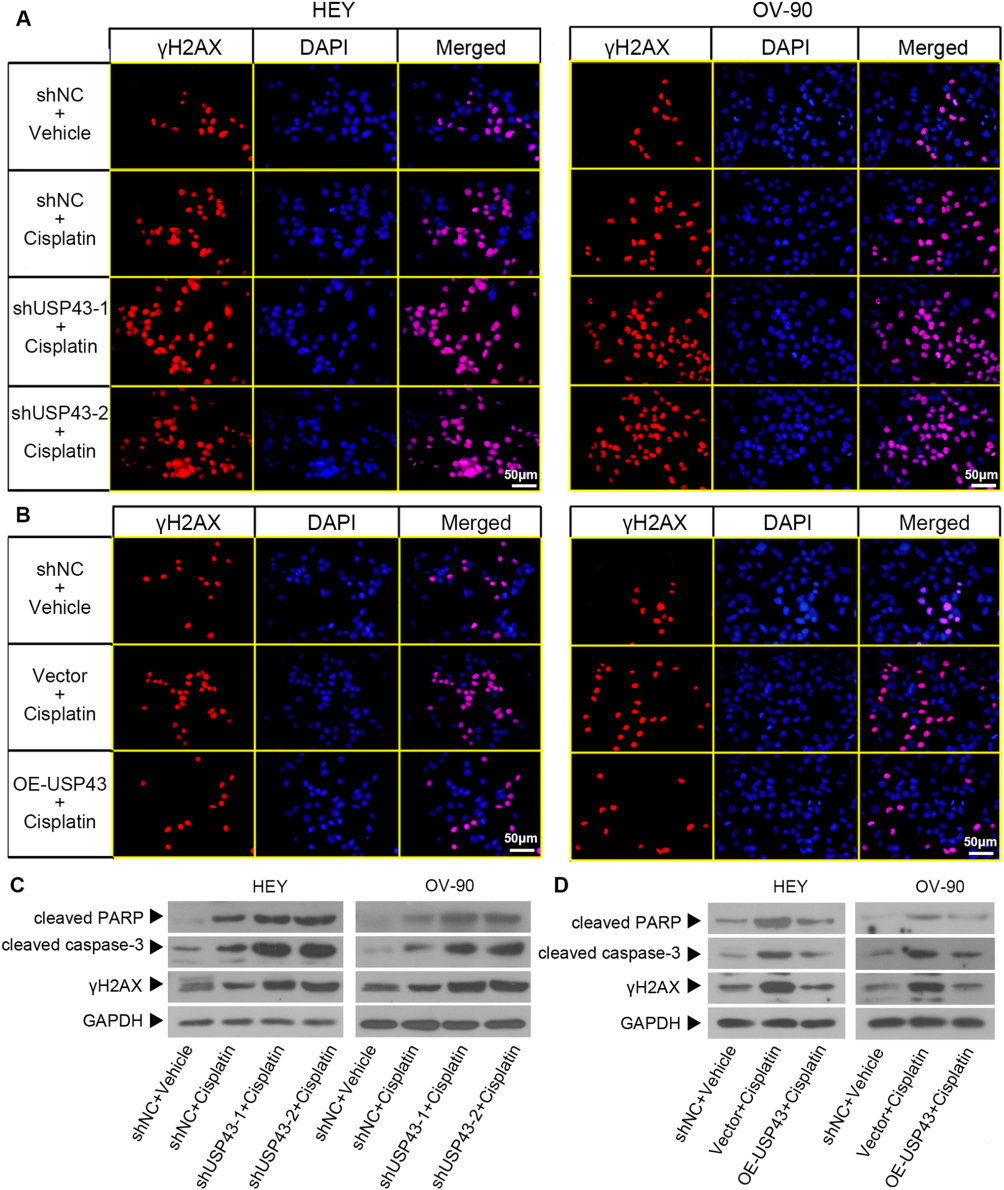
USP43 suppressed cisplatin sensitivity by reducing apoptosis. Stably transfected HEY and OV-90 cells were treated with 10 and 30 µM cisplatin for 48 h, respectively. **A, B** IF staining was used to evaluate the expression of γH2AX (red) in epithelial ovarian cancer. Cell nuclei were stained with DAPI (blue). Scale bar, 50 μm. **C, D** The protein levels of cleaved PARP, cleaved caspase-3 and γH2AX were detected by Western blot

Given that DNA damage is a most prominent mechanism of cisplatin for cancer treatment [24], we further explored the effect of USP43 on cisplatin-induced DNA damage. IF staining showed that the expression of γH2AX induced by cisplatin was increased in USP43-silenced cells, and decreased in USP43-overexpressed cells (Fig. 7A and B). In addition, cisplatin induced an upregulation of cleaved PARP, cleaved caspase-3 and γH2AX expression in EOC cells, and this effect was enhanced in by USP43 silencing and attenuated by USP43 overexpression (Fig. 7C and D). Collectively, these data provided evidences that USP43 repressed the sensitivity to cisplatin of EOC cells in *vitro*. These data suggested that USP43 reduced cisplatin sensitivity in EOC cells through suppressing DNA damage.

### USP43 PPI network and enrichment analysis

**Fig. 8:**
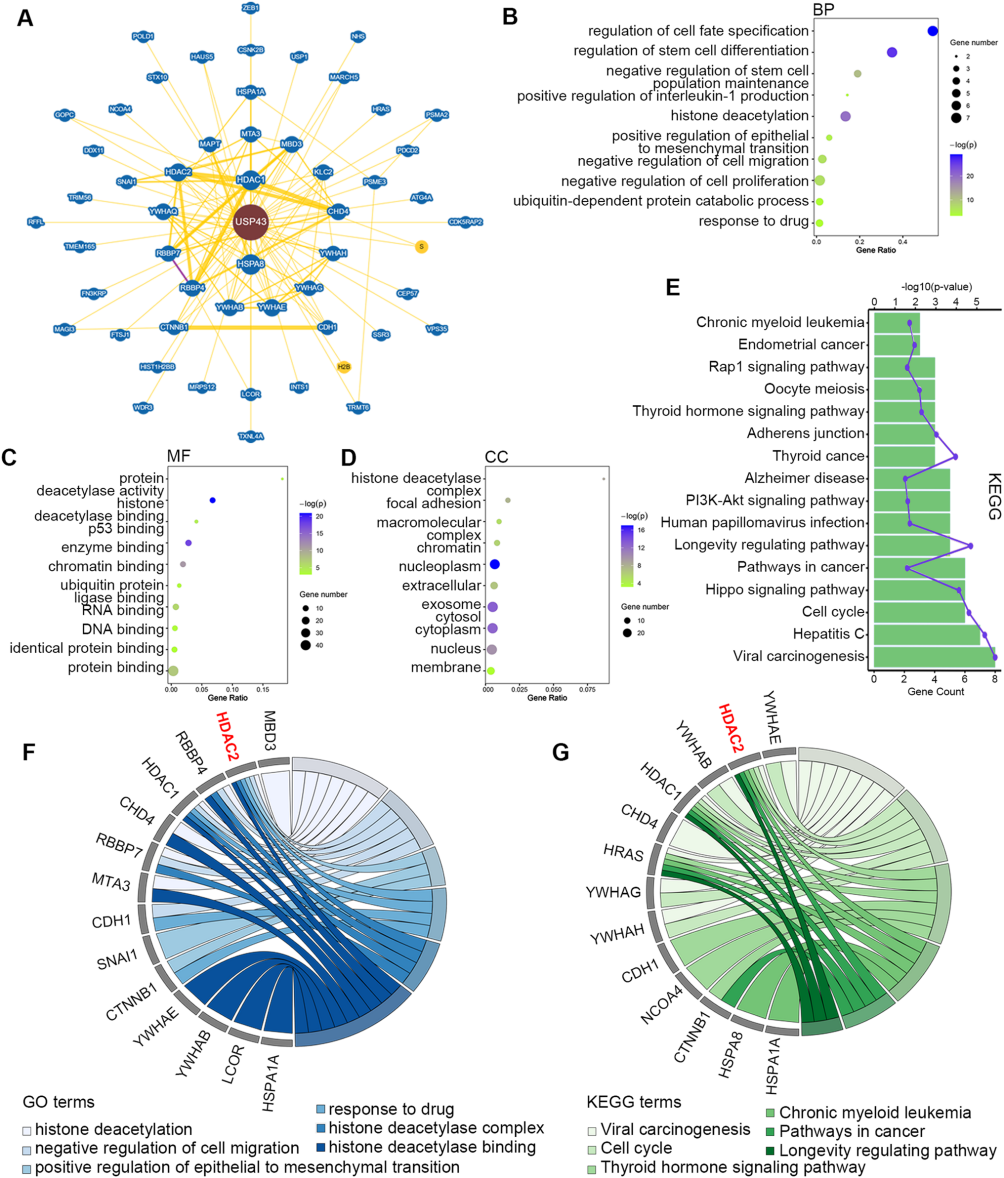
USP43 PPI network and function enrichment analysis. **A** PPI network of USP43 was generated by BioGRID database. **B–D** GO analysis for the predicted genes interacted with USP43. BP, biological process; MF, molecular function; CC: cellular component. **E** KEGG pathway analysis for these genes. **F, G** GO and KEGG enrichment chord diagrams of USP43 related genes. The right side of the circle shows the enriched genes and the color on the left side of the circle represents the different GO terms or KEGG pathways

BioGrid database was used to identify the genes interacted with USP43 and construct a PPI network. A total of 54 interactors were identified as shown in the Fig. 8A. For more effective understanding of these interactors, GO and KEGG pathway enrichment analysis was conducted. In biological process group (Fig. 8B), USP43-related genes were enrichment in regulation of cell fate specification, negative regulation of cell proliferation and migration, ubiquitin-dependent protein catabolic process, and response to drug. For the molecular function group (Fig. 8C), USP43-related genes were mainly enriched in protein deacetylase activity, p53 binding, and ubiquitin protein ligase binding. The cellular component group including histone deacetylase complex, focal adhesion, and macromolecular complex (Fig. 8D). KEGG analysis showed that USP43-related genes had common pathways in PI3K-Akt signaling pathway, Hippo signaling pathway, cell cycle, and pathways in cancer (Fig. 8E). The chord diagram indicated the USP43-related genes were mainly enriched in functions and pathways related to regulation of cancer progression and histone deacetylation (Fig. 8F and G). These findings attracted our attention to the HDAC family. As HDAC2 was reported to be highly expressed in ovarian cancer and associated with poor prognosis, we speculated that a regulatory relation may exist between HDAC2 and USP43.

### USP43 inhibited ubiquitin-mediated degradation of HDAC2 in EOC cells

**Fig. 9:**
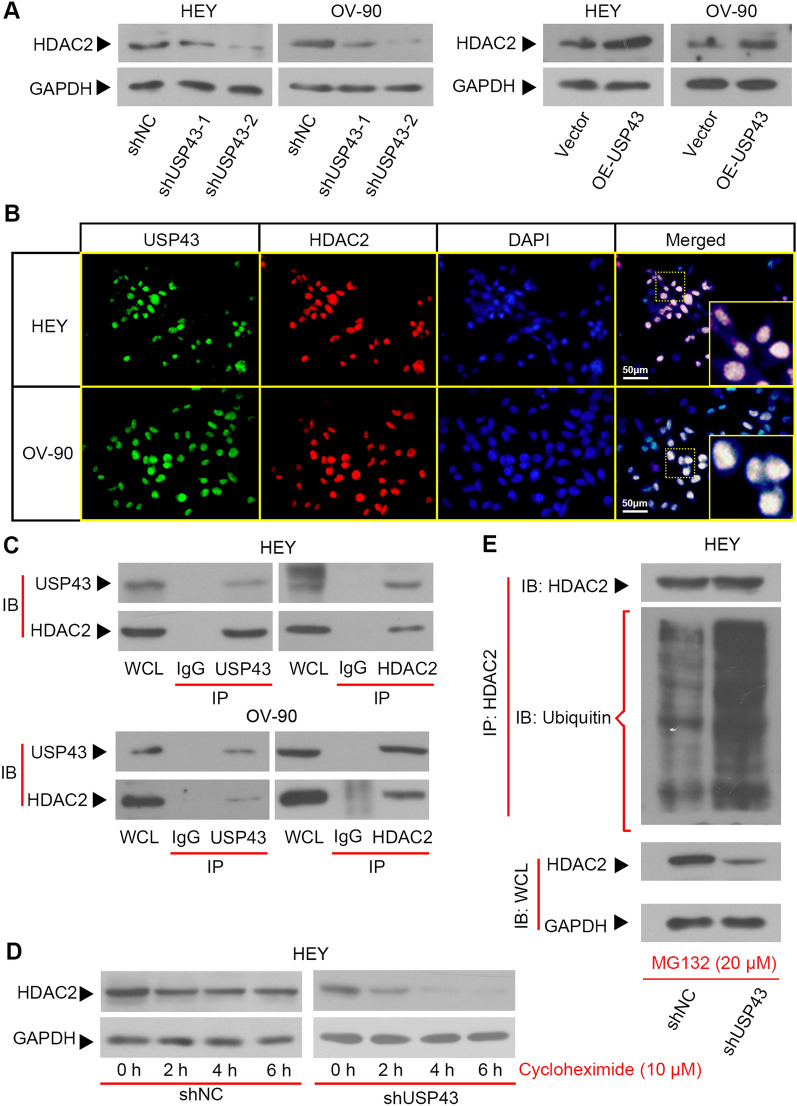
USP43 stabilized HDAC2 through deubiquitylation. **A** Western blot was used to detect the protein level of HDAC2 in HEY and OV-90 cells. **B** IF double staining was used to detect the localization of USP43 (green) and HDAC2 (red). Scale bar, 50 μm. **C** Co-IP assay was used to detect the interaction between USP43 and HDAC2. **D** After treatment with 10 µM cycloheximide for different times, the protein expression level of HDAC2 was observed by Western blot. **E** After treatment with 20 µM MG132, HDAC2 ubiquitination levels were measured by Co-IP

To test our suspicions, we first examined HDAC2 expression in EOC cells with USP43 knockdown or overexpression. We noticed that the protein expression of HDAC2 was downregulated with USP43 silencing and upregulated with USP43 overexpression (Fig. 9A). IF double staining showed that USP43 and HDAC2 were co-expressed in EOC cells (Fig. 9B). The interaction between USP43 and HDAC2 was confirmed by Co-IP assay (Fig. 9C). Cycloheximide, a protein synthesis inhibitor, is often used to inhibit protein synthesis of eukaryotic cells. With cycloheximide treatment, we observed that USP43 knockdown decreased the expression of HDAC2 in HEY cells, indicating that downregulation of USP43 reduced HDAC2 stability (Fig. 9D). To further explore the specific regulatory mechanism of USP43 on HDAC2, the effect of USP43 on HDAC2 ubiquitin levels was examined. As shown in Fig. 9E, the ubiquitin was accumulated in HDAC2 IP products of the shUSP43 cells. These results demonstrated that USP43 diminished HDAC2 degradation via deubiquitylation.

### USP43 impaired cisplatin sensitivity of EOC cells by activating the Wnt/β-catenin signaling pathway through HDAC2

**Fig. 10:**
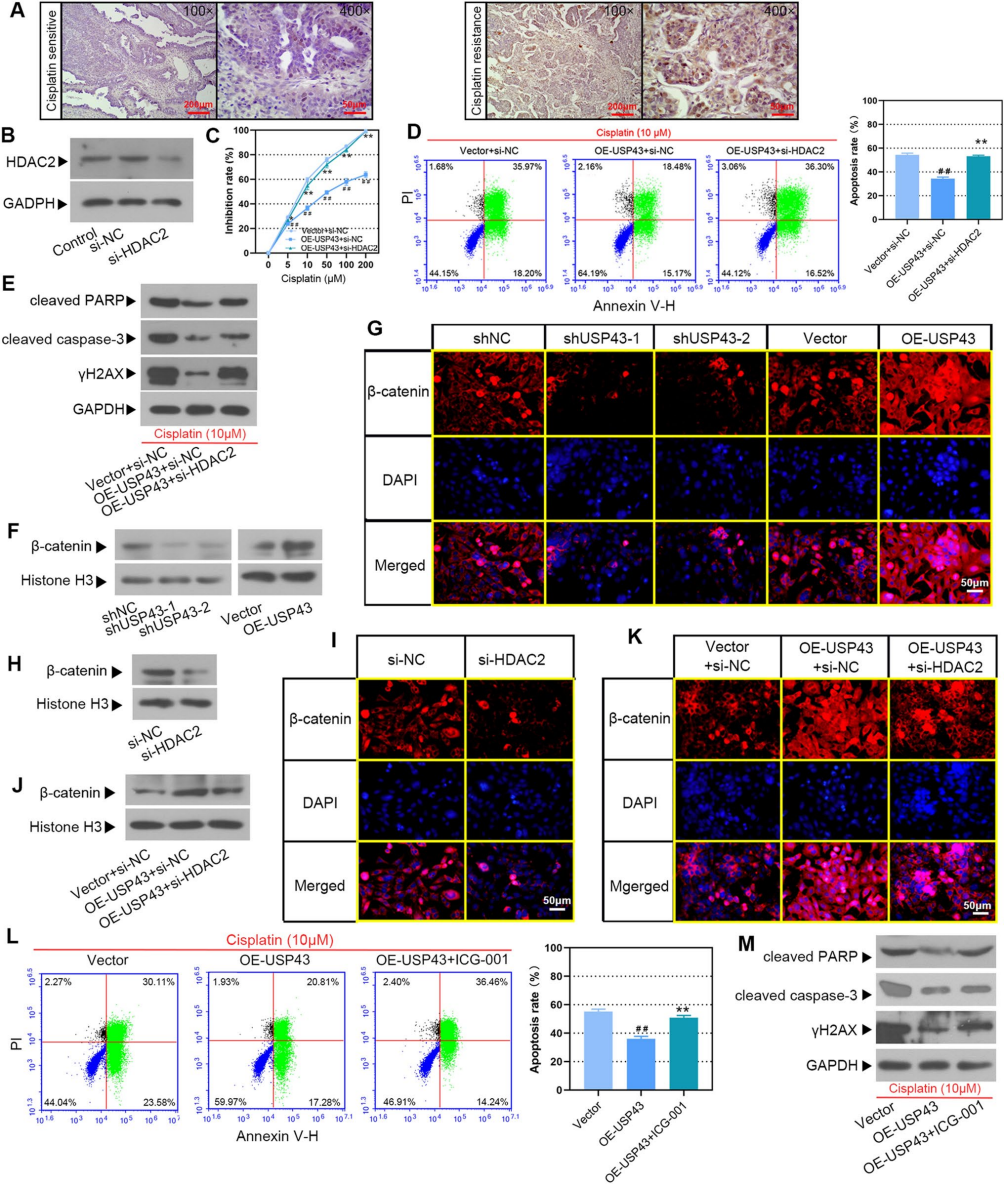
USP43 impaired cisplatin sensitivity of EOC cells by activating the Wnt/β-catenin signaling pathway through HDAC2. **A** Representative images of IHC staining with HDAC2 in cisplatin-sensitive and cisplatin-resistant EOC tissues. Scale bar, 200 μm or 50 μm. HEY cells were transfected with si-HDAC2 or si-NC and 24 h later, **B** the expression of HDAC2 was detected by Western blot. **C–E** USP43 overexpressed HEY cells were treated with 10 µM cisplatin, then **C** cell inhibition rate was detected by CCK-8; **D** cell apoptosis was detected by flow cytometry; **E** Western blot was used to detect the protein levels of cleaved PARP, cleaved caspase-3 and γH2AX. **F** Western blot analysis for β-catenin in HEY cells with or without USP43 knockdown or overexpression. **G** IF staining was used to observe the expression and localization of β-catenin in HEY cells. Scale bar, 50 μm. **H**, **I** HEY cells were transfected with si-HDAC2 or si-NC and 24 h later, β-catenin expression was tested by Western blot and IF staining. Scale bar, 50 μm. **J, K** USP43 overexpressed cells were transfected with si-HDAC2 or si-NC and 24 h later, β-catenin expression was determined by Western blot and IF staining. Scale bar, 50 μm. **L**, **M** USP43 overexpressed cells were treated with 10 µM ICG-001 and cisplatin for 48 h, the cell apoptosis was assessed by flow cytometry and apoptosis-related proteins were analyzed by Western blot. ^*##*^*P* < 0.01 versus Vector + si-NC or Vector; *P < 0.05, **P < 0.01 versus OE-USP43 + si-NC or OE-USP43

As shown in Fig. 10A, HDAC2 expression in the tumor tissues of cisplatin-resistant patients was increased compared to those sensitive to cisplatin. To confirm whether HDAC2 is involved in the USP43-induced reduction of cisplatin sensitivity in EOC cells, USP43 over-expressed cells were transfected with si-HDAC2 or si-NC. Western blot showed that HDAC2 was effectively silenced in Hey cells (Fig. 10B). The overexpression of USP43 attenuated the inhibition of cisplatin on EOC cells, while HDAC2 silencing reversed the result (Fig. 10C). HDAC2 silencing elevated apoptosis rate induced by overexpression of USP43 (Fig. 10D). In addition, the protein levels of cleaved PARP, cleaved caspase-3 and γH2AX induced by USP43 overexpression were further elevated by silencing HDAC2 (Fig. 10E). These results showed that USP43 promoted the sensitivity of EOC cells to cisplatin via HDAC2.

Activation of Wnt/β-catenin signaling pathway is important in the tumorigenesis and development of ovarian cancer [22], and it can be estimated by β-catenin protein levels in the nucleus [25]. As expected, the expression of β-catenin in the nucleus was positively regulated by USP43 (Fig. 10F). IF staining revealed that β-catenin accumulation in the cytoplasm and transport to the nucleus were restrained with USP43 silencing and enhanced with USP43 overexpression (Fig. 10G). The excessive accumulation of β-catenin in the cytoplasm and its translocation to the nucleus are important processes that activate the Wnt/β-catenin signaling pathway. Correspondingly, downregulation of HDAC2 suppressed activation of the Wnt/β-catenin signaling pathway (Fig. 10H, I). Notably, knockdown of HDAC2 diminished the activation of Wnt/β-catenin signaling pathway caused by upregulation of USP43 (Fig. 10J, K). These results indicated that USP43 activated Wnt/β-catenin signaling pathway through HDAC2.

To further determine whether USP43 regulates the sensitivity of EOC cells to cisplatin via the Wnt/β-catenin signaling pathway, EOC cells were co-treated with Wnt/β-catenin signaling pathway inhibitor ICG-001 and cisplatin. USP43 overexpression diminished cell apoptosis induced by cisplatin, and ICG-001 treatment restored this effect (Fig. 10L). The same changes were observed in the expression of cleaved PARP, cleaved caspase-3 and γH2AX (Fig. 10M). Taken together, our results suggested that USP43 impaired the sensitivity of EOC cells to cisplatin by activating the Wnt/β-catenin signaling pathway through HDAC2.

### Knockdown of USP43 inhibited tumor growth and enhanced cisplatin sensitivity of EOCin vivo.

**Fig. 11:**
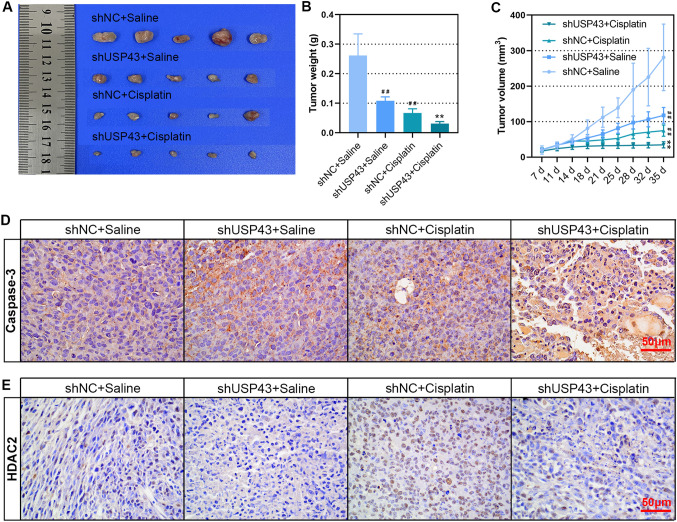
Knockdown of USP43 inhibited tumor growth and enhanced cisplatin sensitivity of EOC *in vivo.* shUSP43 cells or shNC cells (5 × 10^6^) were subcutaneously injected in nude mice, after 7 days, the mice were treated with saline or cisplatin twice every week. After 4-weeks cisplatin-treatment, **A** xenograft tumor was taken out and photographed. **B** The tumor weight was assessed. **C** Tumor volume was examined. **D, E** The expression of cleaved caspase-3 and HDAC2 in subcutaneous tumor tissues was detected by IHC staining. Scale bar, 50 μm. ^*##*^*P* < 0.01 versus si-NC + Saline; **P < 0.01 versus si-NC + Cisplatin

To clarify the effect of USP43 on EOC growth and cisplatin sensitivity in vivo, a subcutaneous xenograft model was constructed. As shown in Fig. 11A–C, the weight and volume of tumors formed by USP43 knockdown cells and treated with cisplatin were significantly reduced. The growth of tumors formed by USP43 knockout cells in cisplatin-treated mice was significantly inhibited. IHC staining for cleaved caspase-3 showed that cisplatin induced apoptosis of EOC cells was aggravated by the downregulation of USP43 (Fig. 11D). Moreover, HDAC2 expression was induced by cisplatin and decreased upon USP43 knockdown (Fig. 11E). Thus, we concluded that knockdown of USP43 inhibited tumor growth and enhanced cisplatin sensitivity of EOC in vivo.

## Discussion

EOC is a common malignant tumor of the female reproductive system, and its mortality rate is the highest among gynecological tumors. Due to the difficulty of early diagnosis, most patients are already at advanced stage when they are diagnosed. Cisplatin is widely used in the treatment of EOC, but cisplatin resistance will affect the treatment effect [26, 27]. In the current study, we demonstrated that USP43 facilitated the malignant behaviors, and decreased the sensitivity of cisplatin to EOC cells. Mechanically, USP43 activated the Wnt/β-catenin signaling pathway through inhibiting ubiquitin-mediated degradation of HDAC2 to induce EOC cell chemoresistance.

USP43 functions as a tumor promoter in multiple cancers. For instance, USP43 promoted cell proliferation, migration and invasion of colorectal cancer [12]. USP43 facilitates breast cancer cell proliferation and cell cycle progression in *vivo* [13]. Our results showed that the expression of USP43 was higher in EOC tissues. Similarly, the protein expression of USP43, analyzed in clinical specimens of the human protein atlas, was barely expressed in normal ovarian tissues and highly expressed in ovarian cancer tissues (Figure S1A). The high expression of USP43 had a lower survival rate (Figure S1B). This also validated the prognostic results of USP43 in EOC tissues. In addition, high expression of USP43 was accompanied by malignant phenotypes in EOC, suggesting that USP43 promotes tumorigenesis in EOC.

Ubiquitination is one of the vital post-translational modifications of proteins, which plays a crucial role in regulation of cell apoptosis [28, 29]. The USP family is one of the most abundant and structurally diverse ubiquitin. USP18 is negatively associated with cell apoptosis in breast cancer [30]. USP39 knockdown induces cell apoptosis via the regulation the Wnt/β-catenin signaling pathway in colorectal cancer [31]. It has been shown that the mechanism underlying cisplatin resistance is related to apoptosis [32–34]. Knockdown of CCAT1 modulates the sensitivity of ovarian cancer cells to cisplatin via promoting cell apoptosis [35]. Our results indicated that USP43 reduced the sensitivity of EOC to cisplatin by decreasing apoptosis. When the DNA damage induced by cisplatin exceeds the DNA repair capacity, cisplatin interferes with the DNA repair mechanisms, resulting in DNA damage and exerts anticancer effects [36, 37]. We revealed that USP43 inhibited cisplatin sensitivity, reduced the number of DNA breaks attacked by cisplatin, and double-strand breaks led to decrease expression of apoptosis proteins. Our results demonstrated that USP43 inhibition of apoptosis affected the sensitivity of EOC to cisplatin.

High-level expression of HDAC2 promotes cancer progression through various signal pathways, including breast cancer [38, 39], non-small cell lung cancer [16], liver cancer [40]. Resistance is a major problem when treating malignancies. Despite advances in drug development, resistance still inevitably occurs over time. HDAC2 is involved in the resistance of tumor cells, inhibition of HDAC2 expression increased the sensitivity of cisplatin in non-small cell lung cancer [41]. Some genes impact the development of malignancies by regulating HDAC2. In ovarian plasmacytosis, USP5 impedes the ubiquitinated degradation of HDAC2, inducing cell proliferation, metastasis and poor prognosis [18]. We indicated that USP43 regulated HDAC2 through deubiquitylation. Moreover, HDAC2 inhibits the expression of apoptotic proteins caspase-3, and PARP in thyroid squamous cell carcinoma [42]. Herein, our findings revealed that USP43 enhances EOC cell chemosensitivity via HDAC2.

Several studies have reported a role for HDAC2 in immune-related modulations [43, 44]. Tumor-infiltrating immune cells are important for cancer treatment and patient prognosis [45]. Laham, Amina Jamal et al. demonstrated the potential role of DYRKs as biomarkers for immunotherapy by analyzing the correlation of its expression with immune infiltrating cells in the tumor microenvironment [46]. Based on these studies, the correlation between HDAC2 expression and the level of immune cell infiltration was analyzed by TIMER. In OV, HDAC2 correlated with tumor purity and CD8+ T cells (Fig. 2SA). Then, we analyzed the relationship between immune cell infiltration and patient survival (Fig. 2SB). Dendritic cells were correlated with prognosis of OV patients. Furthermore, we analyzed the correlation of HDAC2 with immune cell markers (Table S1). Unfortunately, although HDAC2 was associated with some immune infiltrating cells, the correlation coefficients were all less than 0.3. For the immune-related role of HDAC2, it may be exerted indirectly in specific subpopulations of immune infiltrates [47], or through regulation of downstream genes [44].

Abnormal Wnt/β-catenin signaling pathway promotes cancer stem cell renewal, cell proliferation and differentiation, thus exerting essential roles in tumorigenesis and therapy response [48]. The Wnt/β-catenin signaling pathway is an important cellular signaling pathway in cancer and is profoundly influenced by the activity of USPs [49]. Previous studies have reported that USP39 silencing inhibits colon cancer cell growth and metastasis, and induces apoptosis by regulating the Wnt/β-catenin signaling pathway [31]. β-catenin, acting as an intracellular signal transducer in the Wnt/β-catenin signaling pathway, plays a key role in tumorigenesis [21]. Previous studies have shown that β-catenin is abnormally expressed in breast cancer [50], endometrial cancer [51] and cervical cancer [52], which activates the Wnt/β-catenin signaling pathway and initiates the expression of corresponding genes in the nucleus. ARHGAP4 regulates HDAC2 degradation by ubiquitination and enhances the expression of the Wnt/β-catenin signal pathway by regulating β-catenin activation, thus promoting the invasion and metastasis of pancreatic cancer [23]. Wnt/β-catenin signaling pathway plays a vital role in cisplatin resistance in ovarian cancer and participates in the maintenance and propagation of ovarian cancer stem cells [53]. Our result indicated that USP43 activated the Wnt/β-catenin signaling pathway by regulating HDAC2.

DNA methylation and miRNA are critical in the prognostic assessment and potential biomarkers of cancer development [54, 55]. Anuraga, Gangga et al. performed DNA methylation analysis of NEK family members in breast cancer and investigated the relationship between NEK2 and miRNA regulatory network to identify potential prognostic biomarkers in breast cancer [56]. Based on these findings, the Disease Meth web tool (http://bio-bigdata.hrbmu.edu.cn/diseasemeth/) was used to analyze the difference of USP43 methylation level in OV and normal tissues. We found that the methylation level of USP43 was decreased in OV tissues (Fig. 1SC). In addition, miRWalk was used to constructed the miRNA regulatory network with USP43 (Fig. 1SD). Researches have shown that hsa-mir-124-3p and hsa-mir-506-3p were involved in the regulating ovarian cancer development [57, 58]. In addition, studies have shown that USP43 mediates metastasis in breast and colon cancer [12, 59], but it has not been reported in ovarian cancer metastasis or recurrence, and it will be a direction for our investigation in the future.

In conclusion, USP43 impaired cisplatin sensitivity by activating the Wnt/β-catenin signaling pathway via HDAC2 deubiquitylation, thereby promoting EOC progression. USP43 may serve as a potential therapeutic target of EOC.

### Supplementary Information

Below is the link to the electronic supplementary material.

Supplementary material 1 (DOCX 1229.8 kb)

Supplementary material 2 (DOCX 18.6 kb)

## Data Availability

The data used in this study are available from the corresponding author upon reasonable request.
