# Supplementary material for: USP43 impairs cisplatin sensitivity in epithelial ovarian cancer through HDAC2-dependent regulation of Wnt/β-catenin signaling pathway
Source: Apoptosis. 2023 Dec 12;29(1-2):210–28. doi: 10.1007/s10495-023-01873-x (PMC10830728; doi:10.1007/s10495-023-01873-x)
Supplement: Supplementary file 1 — Supplementary material 1 (DOCX 1229.8 kb) [file 10495_2023_1873_MOESM1_ESM.docx]

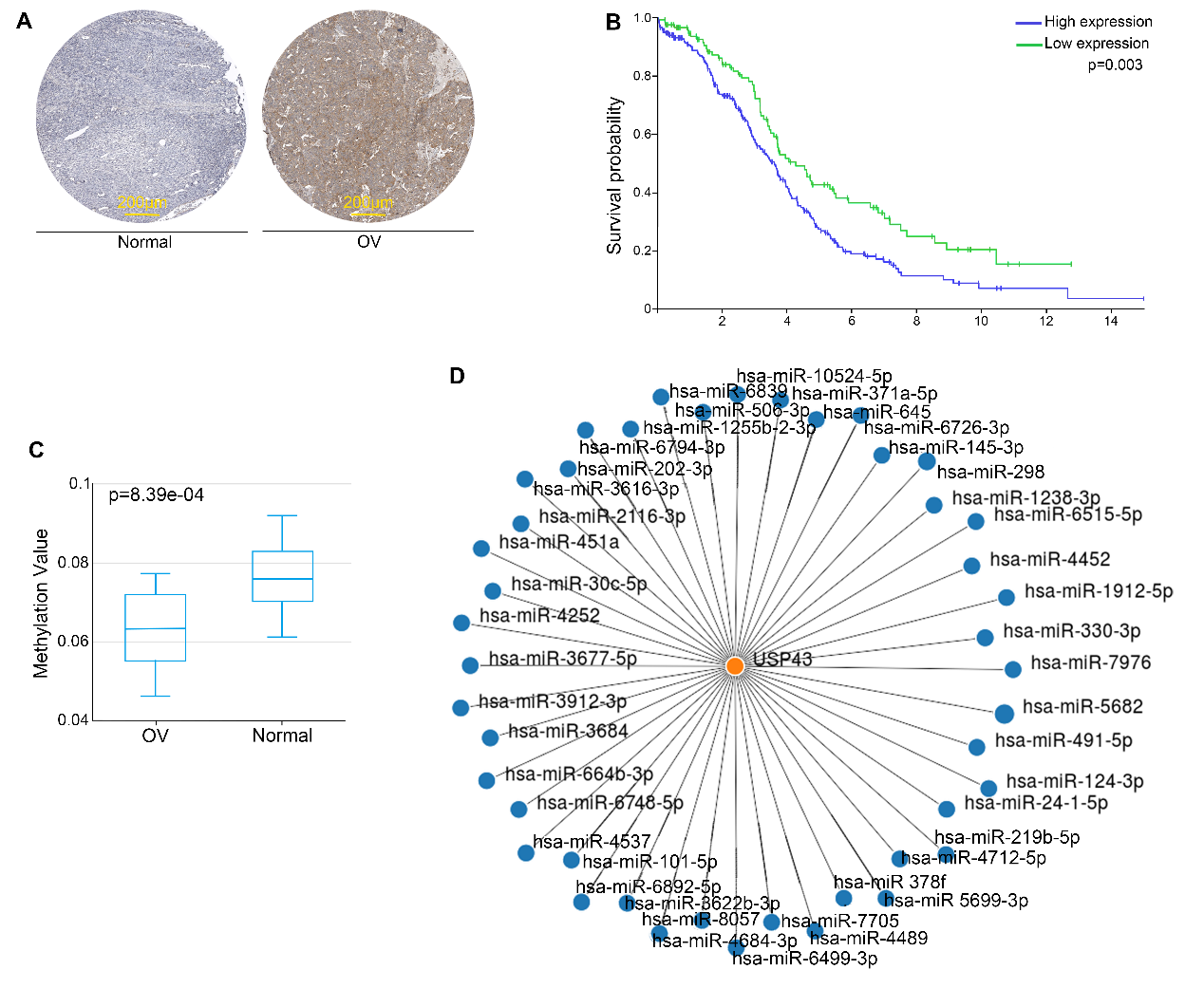


**Fig.S1** (A) IHC stained images show the protein expression of USP43 in clinical specimens of ovarian serous cystadenocarcinoma (OV) and normal tissues in Human Protein Atlas. (B) Association of USP43 expression with survival of OV patients was analyzed by the Human Protein Atlas. (C) The USP43 methylation level in OV and normal tissues was analyzed by DiseaseMeth. (D) The miRNA regulatory network with USP43 constructed by miRWalk.


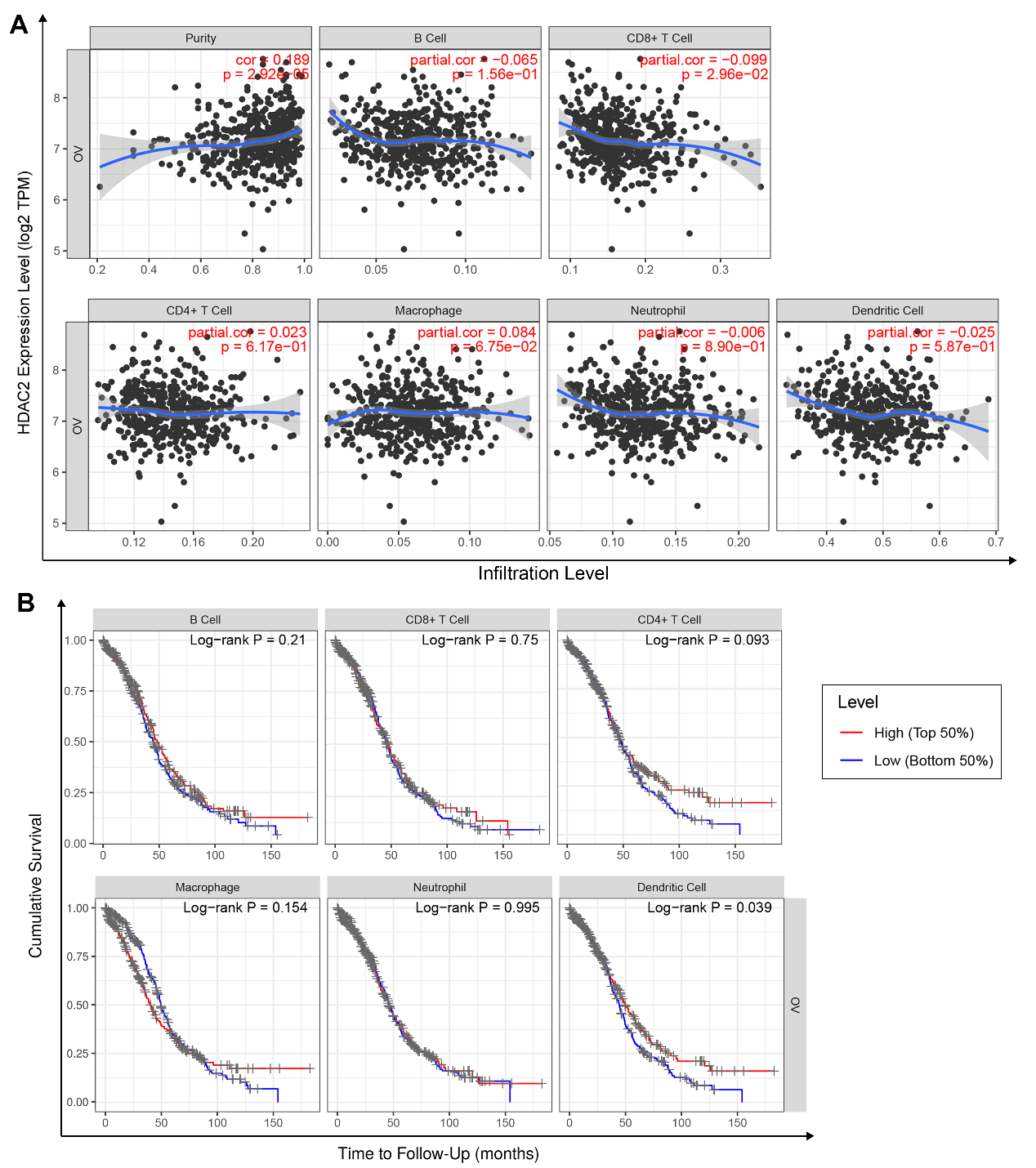


**Fig. S2** (A) The correlation between HDAC2 expression and the level of immune cell infiltration in OV was analyzed by TIMER. (B) The relationship between immune cell infiltration and OV patient survival.
