# Supplementary material for: USP43 impairs cisplatin sensitivity in epithelial ovarian cancer through HDAC2-dependent regulation of Wnt/β-catenin signaling pathway
Source: Apoptosis. 2023 Dec 12;29(1-2):210–28. doi: 10.1007/s10495-023-01873-x (PMC10830728; doi:10.1007/s10495-023-01873-x)
Supplement: Supplementary file 2 — Supplementary material 2 (DOCX 18.6 kb) [file 10495_2023_1873_MOESM2_ESM.docx]

| Description | Gene markers | OV | | | |
| --- | --- | --- | --- | --- | --- |
|  |  | None | | Purity | |
|  |  | Cor | P | Cor | P |
| CD8+ T cell | CD8A | -0.12 | * | 0.017 | 0.794 |
|  | CD8B | -0.012 | 0.833 | 0.112 | 0.0779 |
| B cell | CD19 | 0.111 | 0.053 | 0.125 | * |
|  | CD79A | -0.019 | 0.746 | 0.075 | 0.238 |
| Monocyte | CD86 | -0.219 | *** | -0.078 | 0.222 |
|  | CSF1R | -0.202 | *** | -0.058 | 0.363 |
| TAM | CCL2 | -0.232 | *** | -0.102 | 0.108 |
|  | CD68 | -0.159 | ** | -0.003 | 0.969 |
|  | IL10 | -0.044 | 0.4443 | -0.115 | 0.0698 |
|  | CSF2 | -0.079 | 0.168 | -0.079 | 0.213 |
| M1 | IRF5 | -0.049 | 0.392 | -0.003 | 0.958 |
|  | NOS2 | 0.172 | ** | 0.203 | ** |
|  | PTGS2 | 0.062 | 0.285 | 0.159 | * |
| M2 | CD163 | -0.098 | 0.0872 | 0.072 | 0.258 |
|  | VSIG4 | -0.148 | * | 0.012 | 0.847 |
|  | MS4A4A | -0.139 | * | 0.039 | 0.536 |
| Neutrophils | CEACAM8 | 0.038 | 0.514 | 0.008 | 0.895 |
|  | ITGAM | -0.156 | 0.0648 | 0.015 | 0.812 |
|  | CCR7 | -0.1 | 0.0813 | 0.008 | 0.902 |
| Natrual  Killer cell | KIR2DL1 | -0.128 | * | -0.104 | 0.103 |
|  | KIR2DL3 | -0.161 | ** | -0.153 | 0.159 |
|  | KIR2DL4 | -0.252 | *** | -0.209 | *** |
|  | KIR3DL1 | -0.119 | 0.0377 | -0.102 | 0.108 |
|  | KIR3DL2 | -0.123 | 0.0327 | -0.063 | 0.318 |
|  | KIR3DL3 | -0.084 | 0.143 | -0.072 | 0.26 |
|  | KIR2DS4 | -0.197 | *** | -0.164 | ** |

Table S1. Correlation analysis between HDAC2 and relate genes and markers of immune cells in TIMER.

^*^P<0.05, ^**^P<0.01, ^***^P<0.001.
